# Advances in understanding gray matter pathology in multiple sclerosis: Are we ready to redefine disease pathogenesis?

**DOI:** 10.1186/1471-2377-12-9

**Published:** 2012-03-06

**Authors:** Robert Zivadinov, Istvan Pirko

**Affiliations:** 1 Buffalo Neuroimaging Analysis Center, University at Buffalo, Buffalo, NY, USA; 2 The Jacobs Neurological Institute, University at Buffalo, Buffalo, NY, USA; 3 Department of Neurology, Mayo Clinic, Rochester, Minnesota, USA; 4 Department of Neurology School of Medicine and Biomedical Sciences, Buffalo Neuroimaging Analysis Center, 100 High St., Buffalo, NY 14203, USA

**Keywords:** Multiple sclerosis, Gray matter, Pathology, Meningeal inflammation, Cortical, Subcortical, MRI, Double inversion recovery, Atrophy, Clinical, Treatment

## Abstract

The purpose of this special issue in *BMC Neurology *is to summarize advances in our understanding of the pathological, immunological, imaging and clinical concepts of gray matter (GM) pathology in patients with multiple sclerosis (MS). Review articles by Lucchinetti and Popescu, Walker and colleagues, Hulst and colleagues and Horakova and colleagues summarize important recent advances in understanding GM damage and its implications to MS pathogenesis. They also raise a number of important new questions and outline comprehensive approaches to addressing those questions in years to come. In the last decade, the use of immunohistochemistry staining methods and more advanced imaging techniques to detect GM lesions, like double inversion recovery, contributed to a surge of studies related to cortical and subcortical GM pathology in MS. It is becoming more apparent from recent biopsy studies that subpial cortical lesions in early MS are highly inflammatory. The mechanisms responsible for triggering meningeal inflammation in MS patients are not yet elucidated, and they should be further investigated in relation to their role in initiating and perpetuating the disease process. Determining the role of antigens, environmental and genetic factors in the pathogenesis of GM involvement in MS is critical. The early involvement of cortical and subcortical GM damage in MS is very intriguing and needs to be further studied. As established in numerous cross-sectional and longitudinal studies, GM damage is a better predictor of physical disability and cognitive impairment than WM damage. Monitoring the evolution of GM damage is becoming an important marker in predicting future disease course and response to therapy in MS patients.

## Editorial

Multiple sclerosis (MS) has traditionally been viewed and studied as a chronic inflammatory demyelinating disorder of the central nervous system (CNS) that predominantly involves the white matter (WM). Pathology studies conducted as early as the 19^th ^century have already recognized that MS affects not only the WM but also the gray matter (GM), which somehow got neglected over the years [1]. However, in the last decade, substantial pathological, immunological and imaging evidence confirmed that tissue damage in the GM is a key component of the disease process in MS and that it occurs from the earliest clinical stages [2-5]. During the past few years, the number of studies investigating GM damage in MS has increased exponentially.

This special issue of *BMC Neurology *includes four review articles. One of the primary aims is to provide an educational update not only to general neurologists but also to MS specialists and scientists studying MS by summarizing important recent advances in our understanding of GM damage and its implications to MS pathogenesis. The authors and topics of the articles have been chosen by the guest editors to provide a state-of-the-art review of this rapidly emerging field in MS. The article by Lucchinetti and Popescu focuses on pathology, [4] the article by Walker and colleagues on immunology, [3] by Hulst and colleagues on imaging [2] and by Horakova and colleagues on clinical [5] features of GM involvement in patients with MS.

In the last decade, advanced tissue processing and immunohistochemistry methods, including staining for myelin basic protein (MBP) and proteolipid protein (PLP), [6-8] and more advanced magnetic resonance imaging (MRI) techniques to detect GM lesions, like double inversion recovery (DIR), [9-11] contributed to a surge in studies investigating cortical and subcortical GM pathology in MS.

Although it has been shown that cortical lesions could occur secondary to WM damage in relation to Wallerian degeneration, [12] recent histopathological and MRI studies have demonstrated that cortical demyelination mainly occurs spatially distant from WM pathology [13,14]. It has also been shown that highly inflammatory subpial cortical demyelination and adjacent meningeal inflammation can occur very early on in the disease [15]. Therefore, it is probable that GM could represent an important initial target of the MS disease process.

The histology-based examination of inflammatory infiltration in MS brain tissue includes various markers for T and B cell subsets, dendritic cells, microglia and macrophages [3]. Application of these immunohistochemistry techniques to GM tissue has revealed a contrast with most WM lesions, demonstrating that GM lesions in progressive MS include considerably less inflammation than what is observed in the WM. However, this may simply represent the dynamic evolution of these lesions over the disease course, which is yet to be demonstrated unequivocally [4]. It is becoming apparent from recent biopsy studies that subpial cortical lesions in early MS are highly inflammatory, with intense myelin-laden macrophages and lymphocitic infiltrates similar to active WM lesions, [15,16] whereas in the chronic stages these lesions are markedly less inflammatory, well-demarcated and show oligodendrocyte, axonal and synaptic loss. [6,8,17]

Although a number of different classifications were proposed for distinguishing cortical lesions types over the last decade, [6-8,16] for practical purposes these can be best grouped in 3 subtypes: leukocortical, intracortical and subpial [2-4]. GM lesions have also been described in the cerebellar cortex and hippocampus [18,19]. It has been postulated that the location of a cortical lesion may influence the immune response [3,4]. The amount of inflammation that is present is variable depending on the type of cortical lesion. Lesions that extend through the WM and cortex (leukocortical or type I) have higher counts of inflammatory cells than those that are exclusively intracortical (type II) or subpial (Type III), at least in the chronic stage and based on autopsy material [17,20]. Moreover, there is a close topographic association between subpial lesions and meningeal inflammatory infiltrates [15-17,21]. It is currently believed that meningeal inflammatory aggregates contribute to both cortical demyelination and MS disease progression. Ectopic B-cell follicle-like structures have been reported in the deep sulci of the temporal, cingulate, insula and frontal cortices of early [15] and progressive [22] MS patients and are immunoreactive for Epstein-Barr virus (EBV) [21]. However, the identification of EBV infection of meningeal B-cells and its potential role in MS pathogenesis remains controversial, as these findings have not yet been confirmed by multiple groups. [22]

There is mounting evidence that about 40% of patients with clinically isolated syndrome show cortical lesions on MRI [9]. These data were recently corroborated by histopathological findings [15]. On the other hand more than 80% of progressive MS patients present with cortical lesions in advanced stages of the disease [15,17]. Interesting, in progressive forms of MS, cortical demyelination in the cerebellum is almost universal, affecting over 38% of the entire cerebellar cortex on average [19]. The mechanisms responsible for triggering meningeal inflammation in MS patients are not yet elucidated, and they should be further investigated in relation to their role in initiating and perpetuating the disease process. Determining the role of antigens, environmental and genetic factors for pathogenesis of cortical pathology in MS is critical.

Due to inherent structural differences between GM and WM and as a result of differences in the characteristics of inflammatory infiltrates, GM lesions maintain a normal proton concentration and are not detectable as T2 hyperintense foci like WM lesions [23]. The introduction of DIR in the study of MS played a remarkable step in better recognition of GM lesions [2]. DIR provides excellent distinction between GM and WM by suppressing the signal from normal WM and cerebrospinal fluid [23]. A series of important studies from Geurts et al. [2] and Calabrese et al. [24] established that on DIR the cortical lesions are most frequent in patients with progressive MS, or of male sex or those who have IgG oligoclonal bands. However, it is now evident that detection of cortical lesions in vivo on DIR represents only a limited snapshot of the real cortical and subcortical GM pathology that is present in MS patients, with an average sensitivity of only 18% [25]. Subpial cortical lesions are particularly difficult for DIR to detect. Therefore, although specificity is high in recently established pathologically validated DIR scoring guidelines, sensitivity is very low. [25,26]

MRI and histopathological studies have shown that GM lesions also exist in other non-cortical GM structures such as the thalamus, hippocampus, caudate, putamen, globus pallidum and others [2]. These structures are also affected at the earliest stage, [1] and further progress with disease evolution [27]. Histopathological studies did not show an extensive presence of GM lesions in these structures, when compared to cortical regions [25]. This may suggest that other mechanisms, not yet elucidated, may play an important role in mediating the damage in subcortical GM. The extensive connections between the cortical and subcortical structures, like the thalamus, make these brain structures particularly vulnerable to pathological changes in other areas of the brain [12]. The early involvement of subcortical GM damage in MS is certainly very intriguing and needs to be further studied.

In the last 5 years, numerous cross-sectional and longitudinal studies established that GM damage is a better predictor of physical disability and cognitive impairment than WM damage [5]. Most studies examining this argument used novel imaging techniques that can indirectly assess the extent of GM damage, the most important being a measurement of GM atrophy [2,5]. Therefore, monitoring the evolution of GM damage by various imaging techniques is becoming an important marker in predicting the future disease course and response to therapy in MS patients. A number of current clinical trials examine the effects of immunomodulatory treatments on slowing down GM damage over time.

In conclusion, the review papers by Lucchinetti and Popescu, [4] Walker and colleagues, [3] Hulst and colleagues [2] and Horakova and colleagues [5] represent a comprehensive update on the role and significance of GM damage in MS. They also raise a number of important new questions and outline comprehensive approaches to address those questions in years to come.

## Abbreviations

CNS: Central nervous system; DIR: Double inversion recovery; GM: Gray matter; MBP: Myelin basic protein; MRI: Magnetic resonance imaging; MS: Multiple sclerosis; PLP: Proteolipid protein; WM: White matter

## Competing interests

The authors declare that they have no conflicting interests.

## Disclosures

Robert Zivadinov received personal compensation from Teva Pharmaceuticals, Biogen Idec and EMD Serono for speaking and consultant fees. Dr. Zivadinov received financial support for research activities from Biogen Idec, Teva Neuroscience, Genzyme, Bracco, Questcor Pharmaceuticals and EMD Serono. Dr. Zivadinov serves as Section Editor for *BMC Neurol*.

Dr. Istvan Pirko serves as Clinical Editor for *Nanomedicine: Nanotechnology, Biology and Medicine*; received royalties for publishing in *CONTINUUM *(August 2008); and receives research support from the NIH [#R01NS058698 (PI) and #R01NS060881 (Co-I)].

## Pre-publication history

The pre-publication history for this paper can be accessed here:

http://www.biomedcentral.com/1471-2377/12/9/prepub
